# Genome-Wide Association Study Revealed Candidate Genes Associated with Litter Size, Weight, and Body Size Traits in Tianmu Polytocous Sheep (*Ovis aries*)

**DOI:** 10.3390/biology14101446

**Published:** 2025-10-20

**Authors:** Wenna Liu, Shengchao Ma, Qingwei Lu, Sen Tang, Nuramina Mamat, Yaqian Wang, Wei Hong, Xiangrong Hu, Cuiling Wu, Xuefeng Fu

**Affiliations:** 1 Xinjiang Key Laboratory of Special Species Conservation and Regulatory Biology, International Center for the Collaborative Management of Cross-Border Pest in Central Asia, College of Life Science, Xinjiang Normal University, Urumqi 830054, China; 2 Xinjiang Key Laboratory of Animal Biotechnology, Key Laboratory of Genetic Breeding and Reproduction of Herbivorous Livestock of Ministry of Agriculture and Rural Affairs, Xinjiang Uygur Autonomous Region Academy of Animal Science, Urumqi 830011, China; 3 Zhejiang Sainuo Ecological Agriculture Company, Hangzhou 311300, China

**Keywords:** mixed linear model, reproductive and growth traits, genetic marker, functional enrichment analysis, the influence of genotype on phenotype

## Abstract

**Simple Summary:**

Reproductive and growth traits are important economic characteristics in sheep, regulated by complex molecular mechanisms. Identifying SNPs or candidate genes associated with these traits can provide a theoretical basis for molecular breeding and offer new insights into the genetic foundations of sheep reproduction and growth. Therefore, this study used 483 Tianmu prolific sheep as the research subjects. Through genome-wide association analysis and subsequent bioinformatics analysis, we identified 13, 4, and 7 reliable SNPs associated with litter size, body weight traits, and body size traits, respectively, as well as 18, 8, and 12 candidate genes linked to these traits.

**Abstract:**

Reproductive and growth traits are key economic traits in sheep. This study aims to identify key single nucleotide polymorphisms (SNPs) and candidate genes associated with reproductive and growth traits in Tianmu polytocous sheep through a genome-wide association study (GWAS). The findings are expected to provide both a theoretical foundation for molecular breeding in this breed and novel insights into the genetic basis of ovine reproductive and growth performance. This study took 483 adult Tianmu polytocous ewes as the research subjects, collected their lambing records, measured their phenotypic values of growth traits (3 weight and 11 body size traits), and collected their blood samples for whole-genome resequencing to identify SNPs in the Tianmu polytocous sheep genome. The results identified a total of 9,499,019 (3× coverage) and 27,413,216 (30× coverage) high-quality SNPs in the Tianmu polytocous sheep genome. Subsequently, the association analysis between SNPs and reproductive and growth traits was conducted using a mixed linear model. A total of 92, 66, 18, 28, 6, 42, 3, 3, 6, 1, 12, 3, 22, 8, 6, and 3 SNPs were found associated with litter size at first parity, litter size at second parity, litter size at third parity, litter size at fourth parity, birth weight, weaning weight, body height, withers height, body length, head length, head width, cannon bone circumference, forelimb height, chest girth, chest depth, and withers width, respectively. Further, based on SNP annotation, Gene Ontology (GO) and Kyoto Encyclopedia of Genes and Genomes (KEGG) enrichment analysis, candidate genes associated with the reproductive and growth traits were identified. Among these genes, 11 *LOC*, *DEPTOR*, *GNG12*, *GRM7*, *PTH*, *PTH2R*, *WWOX*, *INHA*, and *NRG3* are candidate genes associated with litter size at first parity or litter size at third parity. These genes are involved in the G protein-coupled receptor signaling pathway, G protein-coupled receptor activity, ovarian tissue development, and hormone secretion. Additionally, *TFRC* and *NTN1* are candidate genes associated with birth weight, while five *UGT1A* and *CASR* are candidate genes associated with weaning weight. These candidate genes are primarily involved in lipid metabolism. Finally, the following genes were identified as candidates associated with specific traits: *DLG2*, *TMEM126A*, and *TMEM126B* with body height; *DSCAM* and *SCN8A* with body length; *BARX1* with cannon bone circumference; four *LOC* genes with forelimb height; *EPHA4* with chest depth; and *MRS2* with withers width.

## 1. Background

Sheep are important agricultural economic animals. With the continuous growth in the consumer market’s demand for mutton [1], improving the reproductive performance of sheep helps expand their population size, thereby providing more market-ready sheep for slaughter. Meanwhile, improving the growth performance of individual sheep helps them reach market weight faster, enhances slaughter rates, and optimizes meat quality, thereby further reducing feeding costs and increasing meat production profits [2,3,4,5,6]. Tianmu polytocous sheep is a new dual-purpose (fur-meat) polytocous sheep population in Zhejiang, China, which was bred through random distant hybridization among four different Hu sheep bloodlines, further followed by three generations of selective breeding and back crossing. This sheep population demonstrates excellent adaptation to local climatic conditions in Zhejiang and has become an important source of mutton production in the region. However, further genetic improvements in both the reproductive and growth performance of Tianmu polytocous sheep through targeted breeding strategies could significantly enhance the economic efficiency of Tianmu polytocous sheep farming operations.

At present, traditional breeding methods have many limitations in improving the reproductive and growth performance of sheep [7]. First, both reproductive (e.g., litter size) and growth traits (e.g., body weight and body size) are quantitative traits. These two categories of traits are regulated by polygenic and non-genetic factors, making it difficult to accurately select superior individuals based solely on phenotypic data [8,9]. Secondly, traditional breeding methods require multiple generations to achieve effective improvements in quantitative traits [7,10]. Traditional breeding is particularly challenging for reproductive trait improvement with generally low heritability [11,12]. Furthermore, pedigree-based inbreeding risks may lead to reduced genetic diversity within the population [10]. Finally, genetic antagonism may exist between different traits, which presents a biological trade-off that is difficult to overcome through traditional breeding methods [13,14]. Collectively, these factors constrain the efficacy of traditional breeding methods in achieving high-efficiency sheep-breeding programs.

Molecular breeding methods (including genomic selection (GS), marker-assisted selection (MAS), and gene editing) have increasingly demonstrated their advantages in improving sheep reproductive and growth performance. First, GS and MAS, based on whole-genome sequencing and high-density SNP chips, can overcome the limitations of traditional phenotypic selection [15,16]. They enable precise breeding by directly targeting key genes or QTLs influencing reproductive and growth performance, significantly improving breeding efficiency [17,18,19,20]. Hence, GS or MAS makes early individual selection possible, shortens the generation interval, and accelerates genetic progress [21].

Identifying SNPs or candidate genes associated with economic traits is a crucial preliminary foundation for applying molecular breeding technologies to economic trait genetic improvement in livestock. With the advancement of genomic analysis technologies, GWAS has emerged as a powerful tool for deciphering the genetic basis of complex traits. By analyzing associations between high-throughput SNP markers and phenotypic data, it enables systematic identification of genetic loci and candidate genes influencing target traits. For instance, Xu et al. conducted separate GWAS studies on five highly prolific sheep breeds (including Wadi, Hu, Icelandic, Finn, and Romanov) and one less prolific breed (Texel). The results revealed multiple candidate genes associated with litter size across different breeds, including *BMPR1B*, *FBN1*, and *MMP2* in Wadi sheep; *GRIA2*, *SMAD1*, and *CTNNB1* in Hu sheep; *NCOA1* in Icelandic sheep; *INHBB*, *NF1*, *FLT1*, *PTGS2*, and *PLCB3* in Finn sheep; *ESR2* in Romanov sheep; and *ESR1*, *GHR*, *ETS1*, *MMP15*, *FLI1*, and *SPP1* in Texel sheep [22]. Gholizadeh and Esmaeili-Fard conducted a GWAS meta-analysis based on 564,377 SNPs from 522 ewes (across six sheep breeds), which further found the role of the *BMPR1B* gene in sheep reproduction [23]. Tuersuntuoheti et al. conducted a GWAS using a mixed linear model for sheep body weight, BHT, BLH, and CGH, identifying 84 candidate genes associated with growth traits (including *BMPR1B*, *HSD17B3*, and *TMEM63C*) [24]. Yang et al. identified three candidate genes associated with body size traits (*SLC9C1*, *VSTM2A*, and *FRG1*) through GWAS in 799 Hulunbuir sheep [25]. However, to date, GWAS research on the reproductive and growth traits of Tianmu polytocous sheep remains insufficient. Therefore, it is necessary to conduct GWAS in the Tianmu polytocous sheep population to identify candidate genes or SNPs associated with reproductive and growth traits.

In summary, this study utilized 483 Tianmu polytocous ewes as research subjects to collect, measure, or calculate the lambing records, birth weight (BWT), weaning weight (WWT), average daily gain (ADG), body height (BHT), withers height (WHT), body length (BLH), head length (HLH), head width (HWH), cannon bone circumference (CBC), forelimb height (FHT), chest girth (CGH), chest depth (CDH), chest width (CWH), and withers width (WWH). Blood samples were also collected from these sheep. Subsequently, routine statistical analysis was conducted on the phenotypic data of the aforementioned reproductive and growth traits, while the blood samples were used for whole-genome resequencing. Building upon the resequencing data, we identified SNPs and performed association analyses between SNPs and lambing number, body weight, and body size traits using a mixed linear model. Significant SNPs were subsequently annotated for gene identification and functional characterization. This study ultimately revealed significant SNPs/candidate genes for lambing performance and growth traits, providing both molecular breeding markers and new genetic insights for sheep reproduction and development research.

## 2. Materials and Methods

### 2.1. Collection of Phenotypic Data and Blood Samples

All 483 Tianmu polytocous ewes were provided by Zhejiang Sainuo Ecological Agriculture Co., Ltd. (Hangzhou, China). These ewes were raised under uniform management conditions, exhibited good growth performance, had similar physical conditions, and were free from any infectious or chronic diseases during the study period. These ewes were studied from birth until the end of their fourth lambing. All the ewes were studied for the same duration, and none of them died during the study. The BWT and WWT of the 483 ewes were measured using a weighing scale at corresponding developmental stages. Based on the WWT and BWT data, the ADG was further calculated. The calculation formula is ADG = (WWT−BWT)/days.

Upon these ewes reaching adulthood (≥ 2 years old), body size traits including BHT, WHT, BLH, HLH, HWH, CBC, FHT, CGH, CDH, CWH, and WWH were measured using standardized measuring sticks and flexible tape measures. Furthermore, the lambing records of all ewes were examined to determine individual litter sizes, with 483 ewes having litter size at first parity (TC1) records, 436 ewes having litter size at second parity (TC2) and TC1 records, 324 ewes having litter size at third parity (TC3), TC2, and TC1 records, and 190 ewes having litter size at fourth parity (TC4), TC3, TC2, and TC1 records. The absence of TC2, TC3, and TC4 data for some individuals is due to these ewes not having lambed multiple times or a lack of actual recorded data.

Finally, all growth trait data were consolidated into Excel 2019 for quality control (quality control criteria: mean ± 3 standard deviations), followed by descriptive statistical analysis (including mean value and standard deviation) and normal distribution analysis performed using SPSS 27.0 and Excel 2019 software. The calculation formula for the coefficient of variation is coefficient of variation = mean/standard deviation. After quality control, a total of 483 records were retained for BWT, BHT, WHT, BLH, HLH, HWH, CBC, FHT, CGH, CDH, CWH, or WWH, along with 473 records for WWT or ADG data.

Next, blood samples were collected from the above 483 Tianmu polytocous sheep. Five mL of blood was collected from each sheep through the jugular vein and stored in EDTA-containing anticoagulant tubes at −20 °C.

### 2.2. Extraction of Genomic DNA, Library Construction, and Sequencing

Blood genomic DNA extraction was carried out using the Blood/Cell/Tissue genomic DNA Extraction Kit (DP304) from Tiangen Biochemical Technology Co., Ltd. (Beijing, China). DNA extraction was conducted in accordance with the instructions of the kit. Electrophoresis experiments were conducted using 1.0% agarose gel (150 V, 30 min) to assess the quality or integrity of genomic DNA. The DNA concentration was determined by NanoDrop 2000 (Thermo Fisher Scientific, Waltham, MA, USA). DNA samples with a concentration greater than 20 ng/μL and OD260/OD280 between 1.7 and 1.9 met the quality standards required for the construction of whole-genome resequencing libraries. The test results show that all 483 DNA samples met the standards. After the DNA product test was qualified, all samples were frozen and stored in a −20 °C refrigerator for future use.

The construction of the 483 libraries was carried out as follows:

DNA samples were fragmented using an M220 ultrasonicator (Covaris, Woburn, MA, USA), and the fragment size (200–400 bp) was verified by a QSep400 Bio-Fragment analyser (Bioptic, Taipei, Taiwan, China);

The sheared DNA fragments were size selected using magnetic beads (≈300 nm, Lnjnbio, Qingpu, Shanghai, China). The specific experimental procedures were performed according to the manufacturer’s instructions.

End repair, A-tailing, and adapter ligation were performed, followed by purification with magnetic beads (≈300 nm, Lnjnbio, Qingpu, Shanghai, China). The specific experimental procedures were performed according to the manufacturer’s instructions.

Following the kit’s instructions, PCR amplification (8–12 cycles) was conducted using KAPA HiFi HotStart DNA Polymerase (Kapa Biosystems, Cape Town, South Africa). The products were then purified with magnetic beads to recover the target fragments, and the final libraries were finally resuspended in elution buffer.

The 483 sequencing libraries were assessed using a Qubit 4.0 fluorometer (Thermo Fisher Scientific, Waltham, MA, USA) for concentration measurement and analyzed with a QSep400 Bio-Fragment Analyser for fragment size distribution. The concentration of library molecules met the minimum concentration required for sequencing loading (15–35 pM), with an insert size greater than 300 bp. After passing these quality controls, the libraries were denatured into single-stranded DNA and processed through a circularization reaction to generate single-stranded circular products. Linear DNA molecules that failed circularization were enzymatically removed. The above experiment was conducted by referring to the instructions provided by the sequencing platform manufacturer (MGI Tech, Yantian, Shenzhen, China). Finally, the circularized libraries were quantified using a Qubit Fluorometer (Thermo Fisher Scientific, Waltham, MA, USA) and sequenced on the DNBSEQ-T7 platform (MGI Tech, Yantian, Shenzhen, China), with 473 samples sequenced at 3× coverage depth and 10 samples at 30× coverage depth.

### 2.3. Preprocessing of Whole-Genome Resequencing Data

First, FastQC 0.12.0 [26] was used to perform quality control on the raw sequencing data, generating clean data free of sequencing errors for downstream analysis. The samples after quality control met the following standards:

1. Removed sequencing adapter/primer sequences from the reads;
2. Trimmed bases at the 3′ end with a quality score (Q) below 20 (i.e., base error rate < 0.01), where Q = −10log(error_ratio);
3. Discarded reads containing more than 5 Ns to filter out reads with excessive ambiguous bases.

Subsequently, the clean data were mapped to the sheep reference genome (GCF_016772045.1_ARS-UI_Ramb_v2.0, Rambouillet sheep) using bwa2-mem (parameters: mem) [27]. The mapped results were sorted using SAMTOOLS [28] and deduplicated using sambamba (parameter: markdup) [29]. GATK 4.5.0.0 [30] was employed to identify SNPs in the resequencing data. High-quality SNPs were obtained through a series of filtering and selection procedures. All samples after quality control met the following standards:

Q20 quality control (removed SNPs with a quality score < 20, i.e., sequencing error rate > 1%);

Filtered SNPs with a missing rate (miss, or SNP deficiency rate) > 0.1;

Filtered SNPs with a minor allele frequency (maf) < 0.05;

Filtered SNPs with per-individual read depth (dp) < 3;

Removed multi-allelic SNP sites.

### 2.4. GWAS

The association analysis between the reproductive/growth traits of Tianmu polytocous sheep and SNPs was conducted using GEMMA 0.98.5 software [31]. Potential candidate SNPs were identified based on their association significance (*p*-value). The screening threshold for SNPs associated with litter size was set at −log_10_P > 5, while the threshold for SNPs associated with body weight and body size traits was −log_10_P > 6. The GWAS was performed using a mixed linear model, incorporating population genetic structure as a fixed effect and individual kinship as a random effect to correct for the influence of population stratification and familial relatedness on the association analysis:

$$y=X_{\alpha }+Z_{\beta }+W_{\mu }+e$$

In this model, y represents the phenotypic trait, X is the design matrix for fixed effects, α denotes the estimated parameters for fixed effects, Z is the SNP design matrix, β indicates SNP effects, W represents the random effects design matrix, μ stands for predicted random individual effects, and e is the random residual following e~N(0, σ^2^e) (where σ^2^e is the residual variance).

Finally, Manhattan plots and Q-Q plots were generated using the CMplot [32] and qqman [33] package in R, respectively. Q-Q plots were determined using all of the common SNPs of 3× and 10× resequencing data.

### 2.5. Bioinformatics Analysis After GWAS

SNP annotation was performed using the ANNOVAR (Released: 2019 Oct 24) software [34]. The SNP annotation results show whether each SNP potentially related to the phenotype is located within or between genes. Subsequently, the DAVID online tool (https://davidbioinformatics.nih.gov/, accessed on 2 January 2025) was employed to conduct GO and KEGG pathway enrichment analyses for candidate genes identified through GWAS. The enrichment results were visualized using the bioinformatics online platform (http://www.bioinformatics.com.cn/, accessed on 10 January 2025). Finally, for the genotype effect of candidate SNPs on reproductive and growth traits, differences between means were analyzed by the *t*-test in GraphPad Prism 8 software. The *p* < 0.05 was considered statistically significant. The online software MSR (http:/www.msrcall.com/Gdical.aspx, accessed on 12 February 2025) was used to analyze the polymorphism information content (PIC), heterozygosity (He), homozygosity (Ho), effective number of alleles (Ne), and Hardy–Weinberg equilibrium (HWE) of SNPs.

## 3. Results

### 3.1. Overview of the Reproductive/Growth Trait Phenotypes of Tianmu Polytocous Sheep

**Figure 1:**
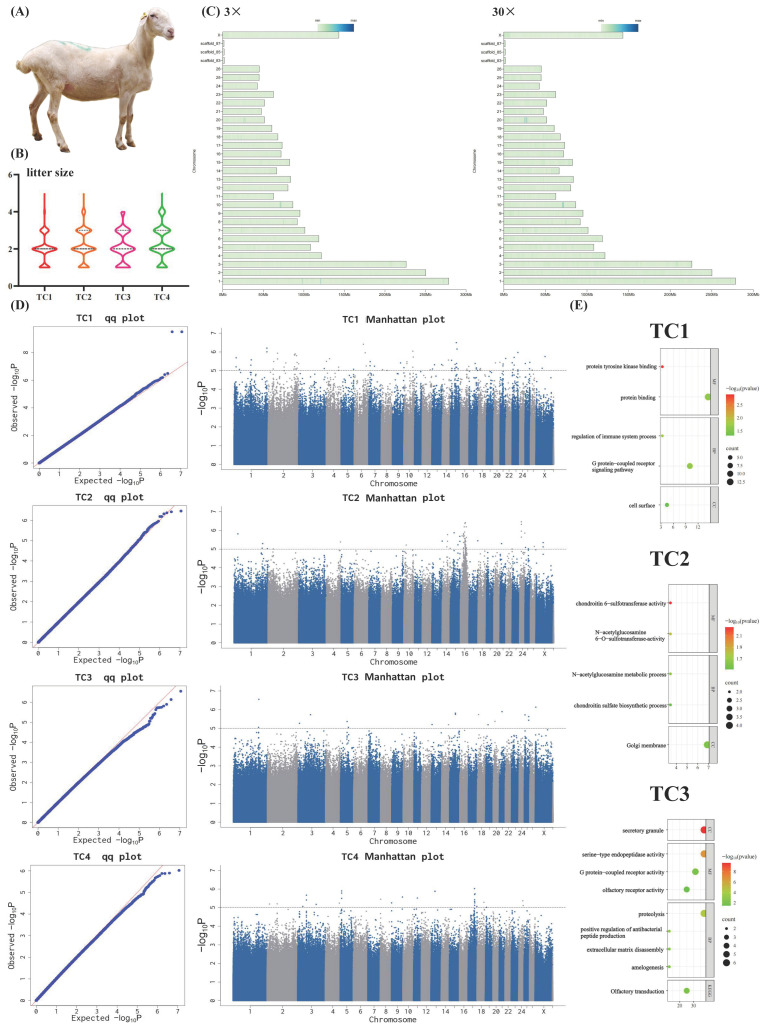
Screening of SNPs/candidate genes associated with litter size traits in Tianmu polytocous sheep. (**A**) Adult Tianmu polytocous ewes. (**B**) Litter size of Tianmu polytocous sheep across different parities. The violin plot shows the distribution of litter sizes in Tianmu polytocous sheep across different parities, with the number of parities on the horizontal axis and the litter size on the vertical axis. (**C**) The distribution of SNPs on chromosomes. The vertical axis corresponds to the chromosome number, and the horizontal axis represents the length of the chromosomes. In the plot, darker colors at specific positions on the chromosomes indicate a higher SNP density at those locations. (**D**) GWAS results for litter size traits. The Manhattan plot displays the statistical significance of the association strength between each tested SNP and the phenotype. The QQ plot illustrates the comparison between the observed distribution of *p*-values from the GWAS association tests and the expected theoretical distribution under the null hypothesis (assuming no true associations). (**E**) The results of functional enrichment analysis of candidate genes associated with litter size traits. The horizontal coordinate of the bubble chart shows the gene ratio. In a bubble plot, the size of the bubbles reflects the number of genes enriched in the pathway/term.

In Tianmu polytocous sheep (Figure 1A), statistical analysis revealed that twin births were the most common across all parities. However, as parity increased, the number of ewes delivering twins showed a declining trend, while those producing triplets and quadruplets demonstrated an upward tendency. Notably, even within the same parity, significant variations in litter size were observed among individual ewes, highlighting substantial individual differences in reproductive performance (Figure 1B and Table S1). These findings clearly indicate that there remains considerable potential for genetic improvement of reproductive traits in Tianmu polytocous sheep.

Statistical analysis revealed that the phenotypic values of each growth trait in Tianmu polytocous sheep conform to or approximately conform to a normal distribution. The mean ± standard deviation of BWT, WWT, and ADG in Tianmu polytocous ewes was 3.31 ± 0.81 kg, 15.85 ± 3.75 kg, and 0.25 ± 0.82 kg, respectively, with corresponding coefficients of variation at 24.48%, 23.66%, and 328.36% (Table S1). Additionally, the mean ± standard deviation of BHT, WHT, BLH, HLH, HWH, CBC, FHT, CGH, CDH, CWH, and WWH in adult Tianmu polytocous ewes was 65.51 ± 2.97 cm, 68.44 ± 3.38 cm, 69.92 ± 3.90 cm, 23.38 ± 1.35 cm, 11.85 ± 0.80 cm, 7.27 ± 0.47 cm, 42.78 ± 1.74 cm, 89.29 ± 6.66 cm, 30.01 ± 2.06 cm, 23.79 ± 12.32 cm, and 17.67 ± 1.42 cm, respectively, with coefficients of variation at 4.54%, 4.93%, 5.58%, 5.76%, 6.75%, 6.48%, 4.07%, 7.46%, 6.87%, 51.80%, and 8.04% (Table S1). These findings indicate that there remains potential for further improvement in the growth traits of Tianmu polytocous sheep.

### 3.2. Sequencing Data Quality Control

This study generated a total of 5.78 Tb of raw sequencing data, with 5.76 Tb remaining after quality control checks were implemented. The Q20 was 99.09 ± 0.00% (mean ± standard deviation), the Q30 was 96.25 ± 0.01% (mean ± standard deviation), and the GC content was 44.14 ± 0.01% (mean ± standard deviation). GC content is defined as the proportion of guanine (G) and cytosine (C) bases relative to the total number of bases in sequencing data. The high Q20/Q30 ratios and balanced GC content distribution demonstrate excellent library preparation and sequencing performance. Collectively, our analyses indicated that all samples met stringent quality standards and were suitable for subsequent in-depth analyses.

### 3.3. Detection and Localization of SNPs

A total of 9,499,019 SNPs were identified in 473 whole-genome resequenced samples (3× coverage), with an average mapping rate of 98.90%. Based on variant detection and quality filtering, 69,552 SNPs were located in coding regions, accounting for 0.73%, among which 29,388 (42.25%) were nonsynonymous variants and 39,682 (57.05%) were synonymous variants. Additionally, 3,489,774 SNPs were located in intronic regions, accounting for 36.74%, while 5,727,314 SNPs were situated in intergenic regions, accounting for 60.29%. In this population, the average number of transitions was 6,829,008, and the average number of transversions was 2,670,011, resulting in a transition/transversion (Ti/Tv) ratio of 2.56. Since the empirical threshold for Ti/Tv is typically >2.1, the higher observed ratio suggests that the majority of the identified SNPs are authentic.

A total of 27,413,216 SNPs were identified from 10 whole-genome resequenced samples (30× coverage), with an average mapping rate of 99.96%. Based on variant detection and quality filtering, 174,049 SNPs (0.63%) were located in coding regions, including 72,097 (41.42%) nonsynonymous variants and 100,838 (57.94%) synonymous variants. Additionally, 9,724,477 SNPs (35.47%) were found in intronic regions, while 16,915,419 SNPs (61.71%) were located in intergenic regions. In this population, the average number of Ti was 19,340,378, and the average number of Tv was 8,072,838, resulting in a Ti/Tv ratio of 2.40. This value suggests that the majority of the identified SNPs are authentic. A total of 9,095,296 SNPs are present in both 3× and 30× resequencing data, and these SNPs were subsequently used for GWAS.

In order to gain a more comprehensive understanding of SNP distribution across the Tianmu polytocous sheep genome, this study analyzed and visualized the SNP distribution on each chromosome (Figure 1C). The SNP density plot revealed that SNPs were distributed across all 26 chromosomes, the X sex chromosome, and scaffold sequences. Furthermore, SNPs exhibited a non-uniform distribution pattern across different chromosomes.

### 3.4. GWAS Results of Litter Size

The Q-Q plots for the four litter size phenotypes show that the expected −log_10_(P) values closely match the observed −log_10_(P) values in the first half of the Q-Q plots, indicating that the models used in the GWAS for TC1, TC2, TC3, and TC4 effectively controlled the false-positive rate, ensuring the reliability of the GWAS results (Figure 1D). The GWAS results further indicated that 92, 66, 18, and 28 SNPs were associated with TC1, TC2, TC3, and TC4, respectively (Tables S2–S5). There is no overlap between SNPs related to TC1, TC2, TC3, and TC4.

The 92 SNPs associated with TC1 are distributed on chromosomes 1, 2, 3, 4, 5, 6, 7, 8, 9, 10, 12, 13, 14, 15, 16, 17, 18, 20, 21, 23, 25, 26, and X (Table S2). Their locations identified a total of 106 genes (Table S2). GO and KEGG enrichment analyses of these 106 genes revealed that 3, 3, 9, 13, and 4 genes were involved in protein tyrosine kinase binding, regulation of the immune system process, the G protein-coupled receptor signaling pathway, protein binding, and the cell surface, respectively (Figure 1E and Table S6).

The 66 SNPs associated with TC2 are distributed on chromosomes 1, 2, 4, 10, 13, 14, 15, 16, 17, 21, 22, 24, 25, 26, and X (Table S3). Their locations identified a total of 62 genes (Table S3). GO and KEGG enrichment analyses of these 62 genes revealed that 2, 2, 2, 4, 2 genes were involved in chondroitin 6-sulfotransferase activity, N-acetylglucosamine 6-O-sulfotransferase activity, the N-acetylglucosamine metabolic process, the Golgi membrane, and the chondroitin sulfate biosynthetic process, respectively (Figure 1E and Table S6).

The 18 SNPs associated with TC3 are distributed on chromosomes 1, 3, 5, 13, 14, 15, 18, 19, 20, 21, 25, and X (Table S4). Their locations identified a total of 19 genes (Table S4). GO and KEGG enrichment analyses of these 19 genes revealed that 6, 6, 6, 2, 2, 2, 5, 4, and 4 genes were enriched or involved in secretory granule (*p* < 0.01 and FDR < 0.01), serine-type endopeptidase activity (*p* < 0.01 and FDR < 0.01), proteolysis (*p* < 0.01 and FDR < 0.01), positive regulation of antibacterial peptide production (*p* < 0.01 and FDR < 0.05), extracellular matrix disassembly (*p* < 0.01 and FDR < 0.05), amelogenesis (*p* < 0.05 and FDR < 0.05), G protein-coupled receptor activity (*p* < 0.05 and FDR < 0.05), olfactory transduction, and olfactory receptor activity, respectively (Figure 1E and Table S6).

The 28 SNPs associated with TC4 are distributed on chromosomes 2, 3, 4, 5, 8, 9, 11, 13, 17, 18, 19, and 24 (Table S5). SNP annotation identified 31 genes (Table S5). GO and KEGG enrichment analyses of these 31 genes revealed that 2 of them (*DNAH9* and *DNAH10*) were enriched in microtubule-related pathways including microtubule-based movement (*p* < 0.05 and FDR < 0.05), dynein light intermediate chain binding (*p* < 0.01 and FDR < 0.01), and dynein intermediate chain binding (*p* < 0.01 and FDR < 0.01) (Table S6).

**Figure 2:**
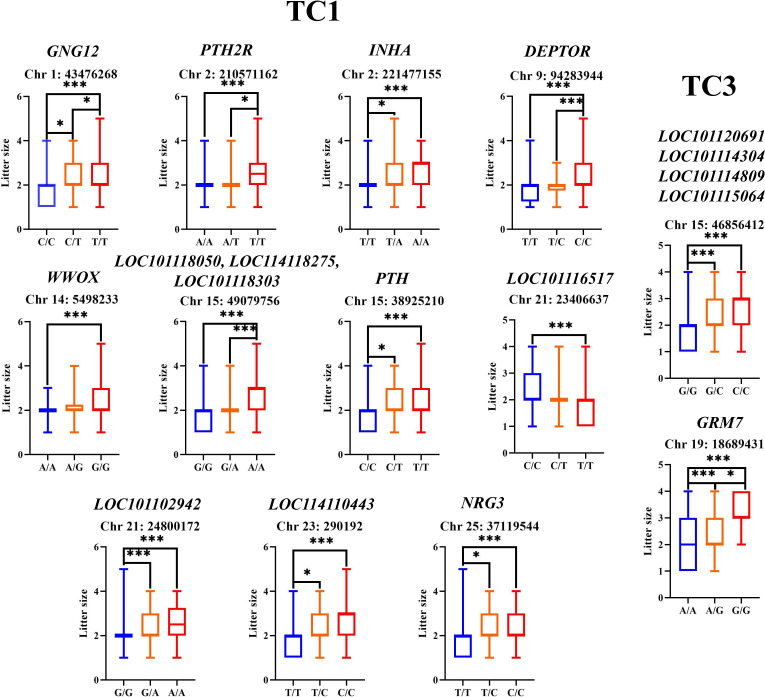
Genotypic distributions of the SNPs for the litter size traits. The genotypes are derived from the resequencing data. The box plot shows the mean values and standard deviations of phenotypic traits corresponding to different genotypes. ***: *p* < 0.01; *: *p* < 0.05.

Finally, based on the SNP annotation results, GO and KEGG enrichment analyses, and the discussion on the functions of candidate genes in the “Discussion” section, we identified candidate genes potentially associated with litter size traits and selected SNPs within these genes for genotypic distribution analysis (Figure 2 and Table S7). Thus, for the TC1 trait, we examined the SNP genotype data for 13 genes located on different chromosomes (Figure 2), including 7 genes (*GNG12*, *DEPTOR*, *LOC101118050*, *LOC114110443*, *LOC114118275*, *LOC101118303*, *LOC101116517*, and *LOC101102942* genes) involved in G-protein-coupling receptor signaling, *WWOX* (Gene ID: 101120431), *PTH2R* (Gene ID: 101120988), *INHA* (Gene ID: 101118082), *PTH* (Gene ID: 101120013), and *NRG3* (Gene ID: 101112537) genes, which respectively encode WW domain containing oxidoreductase, parathyroid hormone receptor 2, inhibin subunit alpha, parathyroid hormone, and neuregulin 3. In the SNP of the *GNG12* gene, individuals with the T/T genotype had a significantly higher (*p* < 0.01) litter size than those with the C/C genotype; in the SNP of the *PTH2R* gene, individuals with the T/T genotype had a significantly higher (*p* < 0.01) litter size than those with the C/C genotype; in the SNP of the *INHA* gene, individuals with the A/A genotype had a significantly higher (*p* < 0.01) litter size than those with the T/T genotype; in the SNP of the *DEPTOR* gene, individuals with the C/C genotype had a significantly higher (*p* < 0.01) litter size than those with the T/T genotype and the T/C genotype; in the SNP of the *WWOX* gene, individuals with the G/G genotype had a significantly higher (*p* < 0.01) litter size than those with the A/A genotype; in the SNPs of the *LOC* genes (*LOC101118050*, *LOC114118275*, and *LOC101118303*) downstream or upstream region, individuals with the A/A genotype had a significantly higher (*p* < 0.01) litter size than those with the T/T genotype and the G/A genotype; in the SNP of the *PTH* gene upstream region, individuals with the T/T genotype had a significantly higher (*p* < 0.01) litter size than those with the C/C genotype; in the SNP of the *LOC101116517* gene upstream region, individuals with the T/T genotype had a significantly higher (*p* < 0.01) litter size than those with the C/C genotype; in the SNP of the *LOC101102942* gene downstream region, individuals with the A/A genotype had a significantly higher (*p* < 0.01) litter size than those with the G/G genotype; in the SNP of the *LOC114110443* gene, individuals with the C/C genotype had a significantly higher (*p* < 0.01) litter size than those with the T/T genotype; in the SNP of the *NRG3* gene, individuals with the C/C genotype had a significantly higher (*p* < 0.01) litter size than those with the T/T genotype.

For the TC3 trait, we examined the SNP genotype data for five genes, including four *LOC* genes implicated in G-protein coupled receptor activity and *GRM7* (Gene ID: 443520), which encodes Glutamate Metabotropic Receptor 7. The genetic contribution analysis of SNPs related to TC3 showed that (Figure 2 and Table S7), in the SNP of the *LOC* genes (*LOC101120691*, *LOC101114304*, *LOC101114809*, and *LOC101115064*), the litter size of individuals with the C/C genotype and those with the G/C genotype was significantly higher (*p* < 0.01) than that of individuals with the G/G genotype; in the SNP of the *GRM7* gene, the litter size of individuals with the G/G genotype was significantly higher (*p* < 0.01) than that of individuals with the A/A genotype and those with the A/G genotype.

### 3.5. GWAS Results of Body Weight Traits

**Figure 3:**
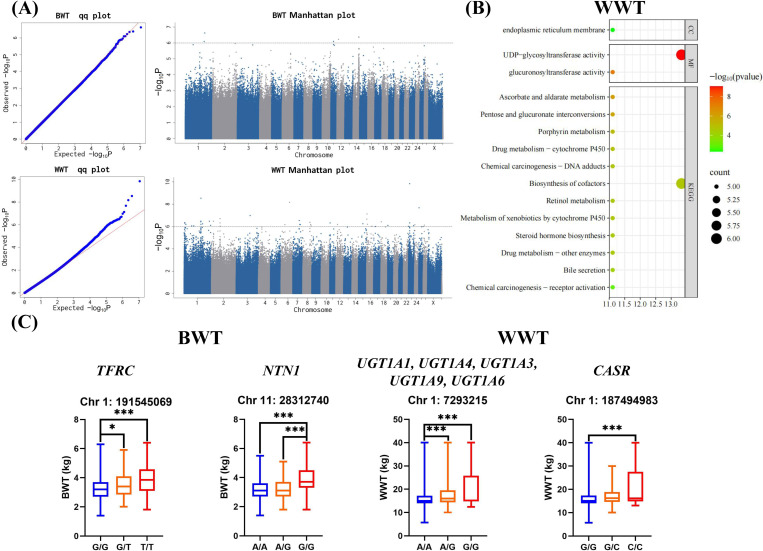
Screening of SNPs/candidate genes associated with body weight traits in Tianmu polytocous sheep. (**A**) GWAS results for body weight traits. (**B**) The results of functional enrichment analysis of candidate genes associated with body weight traits. The horizontal coordinate of the bubble chart shows the gene ratio. In a bubble plot, the size of the bubbles reflects the number of genes enriched in the pathway/term. (**C**) Genotypic distributions of the SNPs for the body weight traits. The genotypes are derived from the resequencing data. The box plot shows the mean values and standard deviations of phenotypic traits corresponding to different genotypes. ***: *p* < 0.01; *: *p* < 0.05.

First, the Q-Q plots of the three body weight phenotypes (Figure 3A and Figure S1) indicated that the models used in the GWAS for BWT and WWT effectively controlled the false-positive rate, ensuring the reliability of the GWAS results for BWT and WWT. The GWAS results further identified 6 SNPs associated with BWT and 42 SNPs associated with WWT (Tables S8 and S9). There is no overlap between SNPs related to WWT and BWT. The SNPs associated with BWT were located on chromosomes 1, 11, 12, and 14, while those associated with WWT were distributed across chromosomes 1, 2, 3, 6, 7, 8, 11, 12, 15, 16, 18, 19, 23, 24, 25, and 26 (Tables S8 and S9). The 72 genes annotated by WWT-associated SNPs were subjected to GO and KEGG enrichment analyses (Table S6). The results showed that 5, 6, 5, 5, 5, 5, 5, 5, 6, 5, 5, 5, 5, 5, and 5 genes were enriched or involved in endoplasmic reticulum membrane, UDP-glycosyltransferase activity (*p* < 0.01 and FDR < 0.01), glucuronosyltransferase activity (*p* < 0.01 and FDR < 0.01), ascorbate and aldarate metabolism (*p* < 0.01 and FDR < 0.01), pentose and glucuronate interconversions (*p* < 0.01 and FDR < 0.01), porphyrin metabolism (*p* < 0.01 and FDR < 0.01), biosynthesis of cofactors (*p* < 0.01 and FDR < 0.01), retinol metabolism (*p* < 0.01 and FDR < 0.01), steroid hormone biosynthesis (*p* < 0.01 and FDR < 0.01), and bile secretion, respectively (Figure 3B and Table S6).

Finally, based on the SNP annotation results, GO and KEGG enrichment analyses, and the discussion on the functions of candidate genes in the “Discussion” section, we identified candidate genes potentially associated with body weight traits and selected SNPs within these genes for genotypic distribution analysis. Thus, for the BWT, we examined the genotype data for two intronic SNPs, the first residing in the *TFRC* gene (Gene ID: 100885768), which encodes a transferrin receptor, and the second in the *NTN1* gene (Gene ID: 105607800), which encodes netrin 1. For the WWT, we examined the genotype data for a SNP that resides in a complex protein-coding region (including *UGT1A1* (Gene ID: 100534644), *UGT1A4* (Gene ID: 100534646), *UGT1A3* (Gene ID: 100534645), *UGT1A9* (Gene ID: 442993), and *UGT1A6* (Gene ID: 100534643) genes) of the genome that, through alternative splicing, encodes several UDP-glucuronosyltransferases, plus an intronic SNP in the *CASR* gene (Gene ID: 101112527) (Table S9), which encodes a calcium sensing receptor. The genetic contribution analysis of BWT-related SNPs showed that (Figure 3C and Table S7), among the SNPs in the *TFRC* genes, individuals with the G/G genotype exhibited significantly higher WWT (*p* < 0.01) compared to those with the A/A genotype and the A/G genotype; among the SNPs in the *CASR* gene, individuals with the C/C genotype had a significantly higher WWT (*p* < 0.01) than those with the G/G genotype.

### 3.6. GWAS Results of Body Size Traits

**Figure 4:**
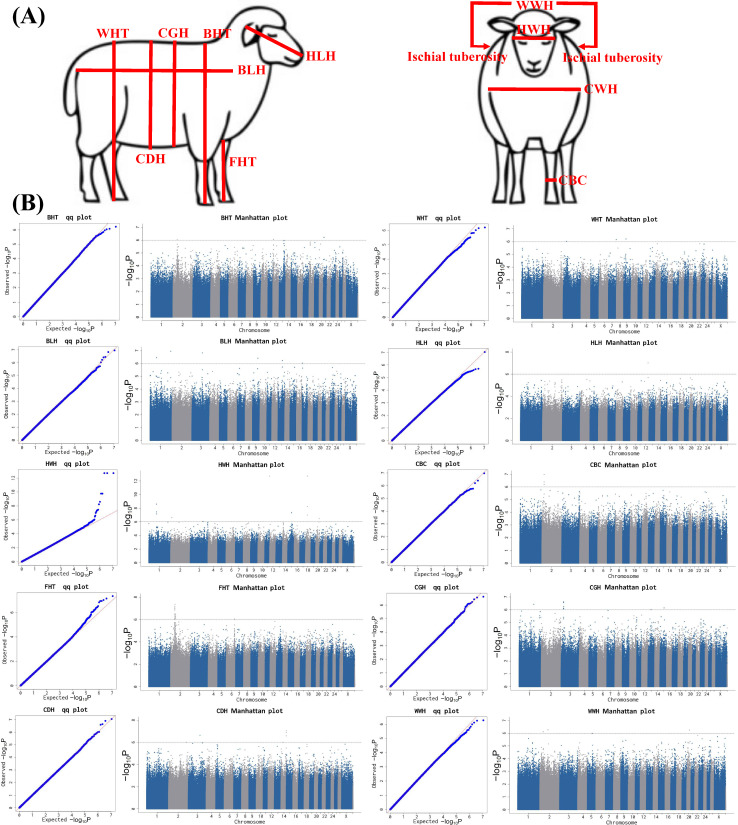
Screening of SNPs/candidate genes associated with body size traits in Tianmu polytocous sheep. (**A**) The 11 body size traits involved in this study. (**B**) GWAS results for body size traits.

The Q-Q plots for the 11 body size traits (Figure 4A) showed that the GWAS models for BHT, WHT, BLH, HLH, HWH, CBC, FHT, CGH, CDH, and WWH effectively controlled the false-positive rate, thereby ensuring the reliability of the GWAS results for these 10 body size traits (Figure 4B, Figure S2). The GWAS results further revealed that 3, 3, 6, 1, 12, 3, 22, 8, 6, and 3 SNPs were significantly associated with BHT, WHT, BLH, HLH, HWH, CBC, FHT, CGH, CDH, and WWH, respectively (Figure 4B, Tables S10–S19).

**Figure 5:**
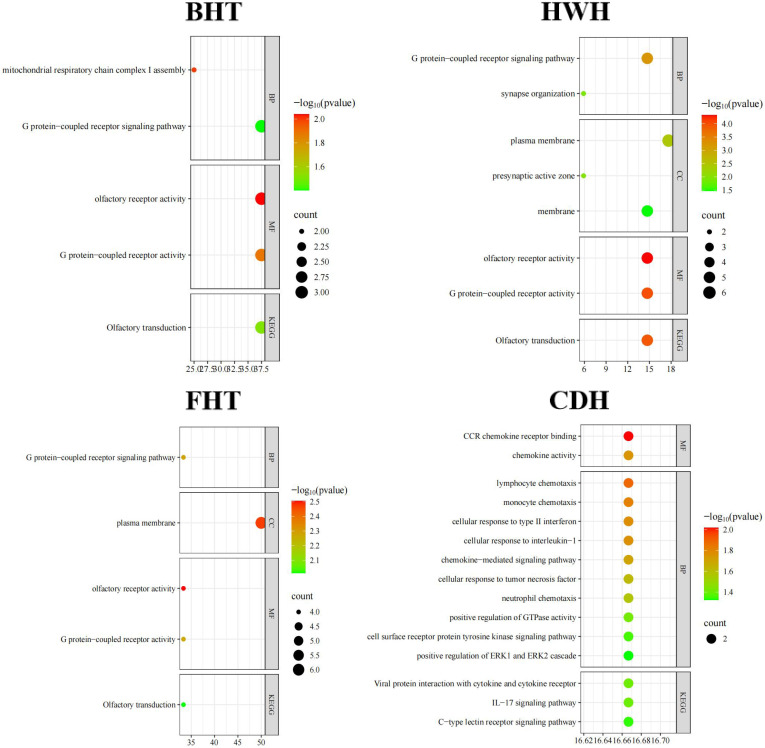
The results of functional enrichment analysis of candidate genes associated with body size traits. The horizontal coordinate of the bubble chart shows the gene ratio. In a bubble plot, the size of the bubbles reflects the number of genes enriched in the pathway/term.

The three BHT-associated SNP reside on chromosomes 2, 12, and 21 (Figure 4B, Table S10). GO and KEGG annotation of the eight candidate genes for the BHT identified by these SNPs (Table S10) revealed connections with the mitochondrial respiratory complex I assembly (*TMEM126A* and *TMEM126B* genes), the G protein-coupled receptor signaling pathway, olfactory receptor activity, G protein coupled receptor activity, and olfactory transduction (Figure 5, Table S6, and Table S10). In comparison, the three WHT-associated SNPs that reside on chromosomes 3, 7, and 9 (Figure 4B and Table S11) identify five candidate genes for this trait, including three genes (*C1D* (Gene ID: 101111532), *FOXN3* (Gene ID: 101114708), and *ZHX2* (Gene ID: 101109810) genes) that encode the C1D nuclear receptor corepressor, forkhead box N3, and zinc fingers and homeoboxes 2.

The six BLH-associated SNPs distributed across chromosomes 1, 3, 9, 13, and 17 (Figure 4B) identify six candidate genes for this trait (Table S12), including *DSCAM* and *SCN8A* genes, both of which are assigned the GO CC term Axon. By contrast, the HLH-associated SNP identified just one candidate gene (*tRNA-Cys*) for this trait (Table S13). Turning to the HWH-associated SNPs distributed on chromosomes 1, 2, 3, 12, 15, 18, and 20 (Figure 4B), annotation of these SNPs identified 14 candidate genes (Table S14). These included an enrichment of genes enriched in the G protein-coupled receptor signaling pathway (*p* < 0.01 and FDR < 0.01), synapse organization (*p* < 0.05 and FDR < 0.05), and olfactory receptor activity (*p* < 0.01 and FDR < 0.01) (Figure 5 and Table S6).

The GWAS of CBC identified three significant SNPs (all located on chromosome 2), and annotation analysis further identified two genes (Table S15). The 22 FHT-associated SNPs are distributed on chromosomes 2 and 6 (Table S16). GO and KEGG enrichment analyses showed that 4, 6, 4, 4, and 4 genes in the 13 genes obtained from the FHT-associated SNP annotation analysis were involved or enriched in the plasma membrane (*p* < 0.05 and FDR < 0.05), olfactory receptor activity, and G protein-coupled receptor activity (Figure 5, Tables S6 and S16).

The eight SNPs associated with CGH are located on chromosomes 1, 3, and 15, and annotation analysis further identified four genes (Table S17). The six SNPs associated with CDH are located on chromosomes 2, 3, and 14 (Table S18). GO and KEGG enrichment analyses showed that *LOC101120186* and *LOC114117787* genes in the 13 genes obtained from the CDH-associated SNP annotation analysis were involved in CCR chemokine receptor binding, lymphocyte chemotaxis, monocyte chemotaxis, cellular response to type II interferon, cellular response to interleukin-1, chemokine activity, the chemokine-mediated signaling pathway, cellular response to tumor necrosis factor, neutrophil chemotaxis, positive regulation of GTPase activity, viral protein interaction with cytokine and cytokine receptor, the IL-17 signaling pathway, the cell surface receptor protein tyrosine kinase signaling pathway, the C-type lectin receptor signaling pathway, and positive regulation of ERK1 and ERK2 cascade (Figure 5, Tables S6 and S18). The three WWH-related SNPs distributed on chromosomes 2 and 20 (Figure 4B, Table S19) identify 10 candidate genes for this trait, including one (*GPLD1* gene (Gene ID: 101119000)) encoding Glycosylphosphatidylinositol Specific Phospholipase D1 and another (*MRS2* gene (Gene ID: 101119262)) encoding magnesium transporter 2 (Table S19).

**Figure 6:**
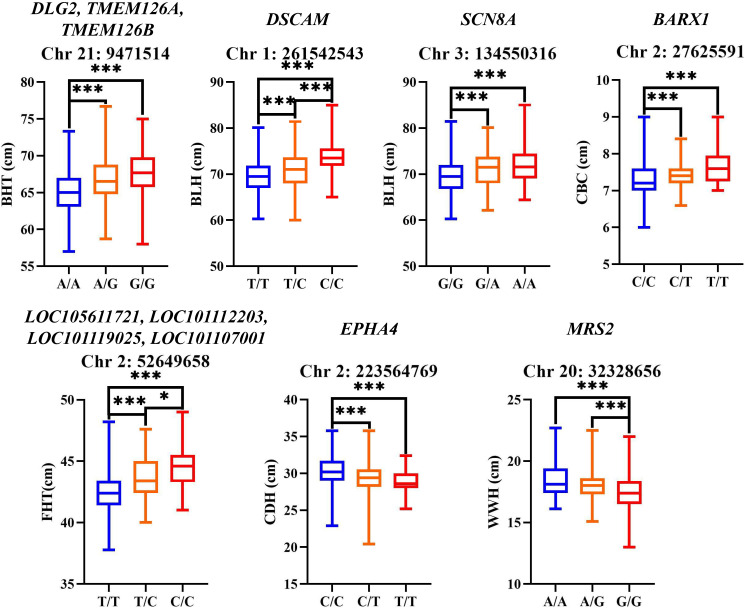
Genotypic distributions of the SNPs for the body size traits. The box plot shows the mean values and standard deviations of phenotypic traits corresponding to different genotypes. ***: *p* < 0.01; *: *p* < 0.05.

Finally, based on the SNP annotation results, GO and KEGG enrichment analyses, and the discussion on the functions of candidate genes in the “Discussion” section, we identified candidate genes associated with body weight traits and selected SNPs within these genes for genotypic distribution analysis. The genetic contribution analysis of BHT-related SNPs showed that (Figure 6 and Table S7), in the *DLG2*/*TMEM126A*/*TMEM126B* genes, G/G genotype individuals had significantly higher (*p* < 0.01) BHT than A/A and A/G genotypes. The genetic contribution analysis of BLH-related SNPs further showed that (Figure 6), in the *DSCAM* gene, individuals with the C/C genotype exhibited a significantly higher (*p* < 0.01) BLH compared to those with the T/T genotype and the T/C genotype. Additionally, the T/C genotype individuals also showed a significantly higher (*p* < 0.01) BLH than the T/T genotype individuals; in the *SCN8A* gene, individuals with the A/A genotype and the G/A genotype had a significantly higher (*p* < 0.01) BLH than those with the G/G genotype. The genetic contribution analysis of CBC-related SNPs further revealed that (Figure 6 and Table S7), in the *BARX1* gene upstream region, individuals with the T/T genotype and the C/T genotype exhibited a significantly higher (*p* < 0.01) CBC compared to those with the C/C genotype. The genetic contribution analysis of FHT-related SNPs showed that (Figure 6 and Table S7), in the *LOC* genes (*LOC105611721*, *LOC101112203*, *LOC101119025*, and *LOC101107001*) upstream region, individuals with the C/C genotype and T/C genotype showed significantly higher (*p* < 0.01) FHT compared to those with the T/T genotype. The genetic contribution analysis of CDH-related SNPs revealed that (Figure 6 and Table S7), in the *EPHA4* gene, individuals with the C/C genotype exhibited significantly higher (*p* < 0.01) CDH compared to those with either the T/T genotype or the C/T genotype. The genetic contribution analysis of WWH-related SNPs showed that (Figure 6 and Table S7), in the *MRS2* gene, individuals with the A/A genotype and A/G genotype showed significantly higher (*p* < 0.01) WWH compared to those with the G/G genotype.

## 4. Discussion

Currently, GWAS has been widely applied in genetic research of livestock and poultry species. Numerous studies have utilized a GWAS approach to identify genomic variants associated with economic traits in livestock and poultry, including reproductive performance, growth performance, disease resistance, etc. [35,36,37]. These findings contribute to a deeper understanding of the genetic mechanisms underlying economic traits in livestock and poultry, providing valuable genetic markers for breed selection and breeding. Therefore, this study aims to uncover SNPs and candidate genes related to reproductive and growth traits in Tianmu polytocous sheep through GWAS, thereby offering critical theoretical foundations for genetic improvement and molecular breeding of Tianmu polytocous sheep.

### 4.1. Identification of Candidate Genes Associated with Litter Size

Based on previous studies, genes such as *BMPR1B*, *BMP15*, and *GDF9* have been widely recognized as major genes influencing sheep reproductive performance [38]. However, this study further identified 204 SNPs significantly associated with litter size (including the TC1, TC2, TC3, and TC4) in the Tianmu polytocous sheep population using GWAS. Based on these 204 SNPs, 218 candidate genes were annotated. These results deepen our understanding of the genetic regulatory mechanisms underlying sheep reproductive performance and provide novel molecular markers affecting sheep litter size. The distribution patterns of these SNPs exhibited certain variations across different parities. SNPs associated with the TC1 were primarily concentrated on chromosomes 2 and 15, those linked to the TC2 were clustered on chromosomes 16 and 14, and those related to the TC4 were mainly located on chromosome 17. This distribution difference may reflect dynamic changes in the genetic regulatory mechanisms of litter size across different parities, suggesting that chromosomes 2, 15, 16, 14, and 17 may be the major chromosomes influencing litter size in Tianmu polytocous sheep.

GO and KEGG enrichment analyses further revealed that among the candidate genes associated with the TC1, genes such as *LOC114110443*, *LOC101116517*, *DEPTOR*, *LOC101118050*, *LOC101102942*, *LOC114118275*, *LOC101118303*, *LOC101117788*, and *GNG12* were involved in the G protein-coupled receptor signaling pathway. For the TC3-related candidate genes, *LOC101120691*, *LOC101114304*, *GRM7*, *LOC101114809*, and *LOC101115064* were enriched in G protein-coupled receptor activity. Previous studies by Wang et al. have demonstrated that the function of G protein-coupled receptors extends to reproductive performance [39]. Steroid hormones such as estrogen and progesterone exert non-genomic regulatory effects on reproduction through G protein-coupled receptor mediation, and G protein-coupled receptor activation is also involved in the development of reproductive systems/tissues. Therefore, we speculate that the aforementioned candidate genes (including *LOC114110443*, *LOC101116517*, *DEPTOR*, *LOC101118050*, *LOC101102942*, *LOC114118275*, *LOC101118303*, *LOC101117788*, *GNG12*, *LOC101120691*, *LOC101114304*, *GRM7*, *LOC101114809*, and *LOC101115064*) may be associated with the reproductive performance of Tianmu polytocous sheep by participating in the G protein-coupled receptor signaling pathway or G protein-coupled receptor activity. Notably, DEPTOR (DEP-domain containing mTOR-interacting protein) is a natural negative regulator of mTOR (mammalian Target of Rapamycin) [40]. The mTOR signaling pathway is activated by luteinizing hormone in ovarian follicles and plays a crucial role in ovulation regulation [41]. These findings further support that the *DEPTOR* gene is an important candidate gene associated with the reproductive performance of Tianmu polytocous sheep.

Finally, among the candidate genes associated with the TC1, we also noted that some SNPs were annotated to genes such as *PTH*, *PTH2R*, *WWOX*, *NRG3*, and *INHA*. The *PTH* gene encodes parathyroid hormone protein, while PTH2R belongs to the G protein-coupled receptor family and serves as the receptor for parathyroid hormone. As previously mentioned, the G protein-coupled receptor is closely linked to animal reproductive performance. Studies have reported that altered expression levels of *PTH* and *PTH2R* genes may be associated with ovarian disorders, such as elevated serum parathyroid hormone concentrations in polycystic ovary syndrome patients [42]. Additionally, PTH2R protein has been implicated in the proliferation and migration of ovarian cancer cells [43]. The WW domain-containing oxidoreductase (*WWOX*) gene has been shown to down-regulate the expression of cyclin E-CDK2 and cyclin D1-CDK4, affecting the cell cycle of ovarian cancer stem cells. Alternatively, it may up-regulate Wnt-5α, JNK, and caspase-3 mRNA expression, thereby promoting apoptosis in ovarian cancer stem cells [44]. The *NRG3* gene, a member of the neuregulin family, encodes an extracellular protein ligand (neuregulin 3) [45]. This protein can bind to ErbB4, participating in ErbB signaling, which is associated with ovarian tumorigenesis [46]. Based on ovarian tissues/cells playing a decisive role in animal reproductive capacity and the above findings, we hypothesize that *PTH*, *PTH2R*, *WWOX*, and *NRG3* may also be important candidate genes associated with the reproductive performance of Tianmu polytocous sheep. Their regulatory effects may be mediated through the growth, development, and pathological changes of ovarian tissues/cells. Additionally, inhibin alpha (INHA), a member of the transforming growth factor-β superfamily, plays a crucial role in regulating the reproductive axis and affects all reproduction-related events [47]. Cui et al. further demonstrated through expression and SNP analyses that the *INHA* gene is a potential candidate gene for improving reproductive traits in chickens [48]. Therefore, *INHA* may also be an important candidate gene regulating the reproductive performance of Tianmu polytocous sheep.

### 4.2. Identification of Key Candidate Genes Associated with Body Weight Traits

Body weight in livestock is primarily associated with the growth and development of muscle and skeletal tissues, as well as the deposition of body fat. The regulation of body weight involves multiple levels, including genetics, physiology, biochemistry, etc. GWAS lays the foundation for understanding the genetic regulatory mechanism of body weight traits in Tianmu polytocous sheep. Among the 10 candidate genes (*TFRC*, *LOC114109072*, *NTN1*, *LOC105601981*, *LOC105601982*, *LOC114118161*, *SIGLEC5*, *TRNAC-GCA-182*, *ZNF175*, and *LOC101110828*) associated with BWT in Tianmu polytocous sheep, 2 genes (*TFRC* and *NTN1*) have been previously reported to be involved in lipid metabolism or energy metabolism. Specifically, the *TFRC* (Transferrin Receptor) gene regulates iron metabolism. Iron deficiency may affect glucose transport, mitochondrial function, and adipocyte differentiation, and has been linked to obesity and insulin resistance [49]. The *NTN1* (Netrin-1) gene encodes a neural guidance molecule. Meanwhile, recent studies by Wang et al. in pigs revealed that the *NTN1* gene influences intramuscular fat content by modulating the expression of myogenic regulatory factors [50]. Additionally, Mentxaka et al. found that Netrin-1 promotes visceral adipose tissue inflammation in obese individuals and is associated with insulin resistance, suggesting functional similarities with the *TFRC* gene [51]. Alterations in lipid metabolism can affect fat deposition patterns in animals, thereby influencing body weight, while energy metabolism broadly impacts growth and development. Therefore, we speculate that *TFRC* and *NTN1* may be important candidate genes associated with the BWT of Tianmu polytocous sheep.

Among the 72 candidate genes associated with WWT, we identified that *UGT1A1*, *UGT1A4*, *UGT1A3*, *UGT1A9*, and *UGT1A6* were enriched or involved in endoplasmic reticulum membrane, steroid hormone biosynthesis, and bile secretion. It is well established that the endoplasmic reticulum serves as the primary site for lipid synthesis in animals, while bile facilitates the breakdown of large lipid molecules in the digestive tract, generating smaller lipid molecules that can be utilized for fat synthesis and deposition. Additionally, steroid hormones play a critical role in animal growth and development. Furthermore, previous studies have reported that the *CASR* gene exerts an anti-lipolytic effect by suppressing cAMP signaling [52,53]. Given the established importance of lipid metabolism in regulating body weight changes in animals, we propose that *UGT1A1*, *UGT1A4*, *UGT1A3*, *UGT1A9*, *UGT1A6*, and *CASR* may serve as key candidate genes influencing the WWT of Tianmu polytocous sheep. These genes likely regulate BWT by modulating lipid metabolism.

### 4.3. Identification of Candidate Genes Associated with Body Size Traits

The body size of livestock is primarily determined by skeletal development, while factors such as muscle development and fat deposition also influence their phenotypic traits. Further, body morphological characteristics are regulated by complex molecular mechanisms, with different body size traits potentially governed by distinct major genes. Based on annotation of GWAS SNP data, we identified eight candidate genes (*HRCT1*, *LOC101107001*, *LOC101112203*, *LOC101119025*, *SPAAR*, *DLG2*, *TMEM126A*, and *TMEM126B*) associated with BHT in Tianmu polytocous sheep. Among these candidate genes, the *DLG2* gene has been linked to new skeletal tissue formation [54]. Meanwhile, the *TMEM126A* and *TMEM126B* genes encode mitochondrial inner membrane proteins, with previous studies indicating their involvement in mitochondrial function regulation [55,56]. Abnormal energy metabolism in mitochondria may impair skeletal or muscle growth, thereby affecting body size traits. Thus, we consider that *DLG2*, *TMEM126A*, and *TMEM126B* might be important candidate genes associated with BHT. Among the candidate genes (*DSCAM*, *SCN8A*, *LOC121820349*, *TRNAC-GCA-141*, *LOC114117597*, and *STK35*) associated with BLH in Tianmu polytocous sheep, the *DSCAM* gene has been reported to cause skeletal growth abnormalities (e.g., scoliosis) in humans when mutated [57,58], which could similarly influence BLH in livestock. In addition, the *SCN8A* gene encodes a voltage-gated sodium channel primarily involved in neuronal electrical signaling [59]. In humans, *SCN8A* mutations may lead to growth retardation [60], thereby affecting body size. Clearly, *DSCAM* and *SCN8A* might be important candidate genes associated with the BLH of Tianmu polytocous sheep.

Among the candidate genes (*BARX1* and *PTPDC1*) associated with CBC in Tianmu polytocous sheep, the *BARX1* gene belongs to the homeobox gene family and is expressed in joints and articular cartilage during limb development [61]. The *BARX1* gene controls the differentiation of skeletal precursor cells into chondrocytes or joint cells [62]. Since CBC is an important indicator of limb development in livestock, these findings suggest that *BARX1* may be an important candidate gene associated with this trait in Tianmu polytocous sheep. Functional enrichment analysis of candidate genes (*FAM221B*, *HRCT1*, *LOC101107001*, *LOC101112203*, *LOC101119025*, *LOC105611721*, *SPAAR*, *TMEM8B*, *FAM214B*, *UNC13B*, *GNAQ*, *LOC114115249*, and *ZNF518B*) related to FHT revealed that *LOC105611721*, *LOC101112203*, *LOC101119025*, and *LOC101107001* were enriched in the G protein-coupled receptor signaling pathway and G protein-coupled receptor activity. Previous studies have demonstrated that the G protein-coupled receptor plays a crucial role in bone metabolism and skeletal remodeling [63,64]. Therefore, it is reasonable to hypothesize that these genes (*LOC105611721*, *LOC101112203*, *LOC101119025*, and *LOC101107001*) may be associated with FHT in Tianmu polytocous sheep by participating in GPCR-mediated signaling pathways. Among the candidate genes (*EPHA4*, *ABCD2*, *CIAPIN1*, *COQ9*, *DOK4*, *LOC101120186*, *LOC114117787*, *LOC114117882*, *POLR2C*, *LOC101110699*, *LOC101117358*, *LOC114118101*, and *LOC121816397*) associated with CDH, the *EPHA4* gene encodes the Ephrin Receptor A4 protein. The EphA4 receptor acts as a novel negative regulator of osteoclast activity [65], and studies by Ting et al. showed that EphA4, as an effector of Twist1, guides osteoprogenitor cells during cranial bone growth and suture closure [66]. Finally, among the candidate genes (*LOC114118859*, *RHOBTB2*, *NDUFB6*, *SMIM27*, *TOPORS*, *TRNAS-GGA-54*, *GPLD1*, *LOC114109798*, *LOC121817383*, and *MRS2*) linked to WWH, the MRS2 protein is involved in mitochondrial magnesium transport [67]. Magnesium ions are essential minerals for skeletal development [68]. Therefore, based on the above reports, we speculate that the *EPHA4* and *MRS2* genes are important candidate genes affecting CDH and WWH, respectively.

## 5. Conclusions

Based on GWAS, this study identified SNPs and important candidate genes associated with reproductive and growth traits in Tianmu polytocous sheep. The findings not only contribute to the genetic improvement of Tianmu polytocous sheep but also provide new insights into the genetic architecture of complex traits in sheep.

## Data Availability

The original contributions presented in the study are included in the article; further inquiries can be directed to the corresponding authors.

## Supplementary Materials

The following supporting information can be downloaded at https://www.mdpi.com/article/10.3390/biology14101446/s1. Figure S1. GWAS results for ADG. Figure S2. GWAS results for CWH. Table S1. Descriptive statistical results of phenotypic values of reproductive and growth traits. Table S2. Basic information of SNPs/candidate genes significantly associated with TC1. Table S3. Basic information of SNPs/candidate genes significantly associated with TC2. Table S4. Basic information of SNPs/candidate genes significantly associated with TC3. Table S5. Basic information of SNPs/candidate genes significantly associated with TC4. Table S6. The GO and KEGG enrichment analysis results of SNPS significantly related to phenotypes. Table S7. Analysis results of the genotypic distributions of SNPS significantly related to phenotypes. Table S8. Basic information of SNPs/candidate genes significantly associated with BWT. Table S9. Basic information of SNPs/candidate genes significantly associated with WWT. Table S10. Basic information of SNPs/candidate genes significantly associated with BHT. Table S11. Basic information of SNPs/candidate genes significantly associated with WHT. Table S12. Basic information of SNPs/candidate genes significantly associated with BLH. Table S13. Basic information of SNPs/candidate genes significantly associated with HLH. Table S14. Basic information of SNPs/candidate genes significantly associated with HWH. Table S15. Basic information of SNPs/candidate genes significantly associated with CBC. Table S16. Basic information of SNPs/candidate genes significantly associated with FHT. Table S17. Basic information of SNPs/candidate genes significantly associated with CGH. Table S18. Basic information of SNPs/candidate genes significantly associated with CDH. Table S19. Basic information of SNPs/candidate genes significantly associated with WWH.
