# Supplementary figures and images for: Genome-Wide Association Study Revealed Candidate Genes Associated with Litter Size, Weight, and Body Size Traits in Tianmu Polytocous Sheep (Ovis aries)
Source: Biology (Basel). 2025 Oct 20;14(10):1446. doi: 10.3390/biology14101446 (PMC12561659; doi:10.3390/biology14101446)

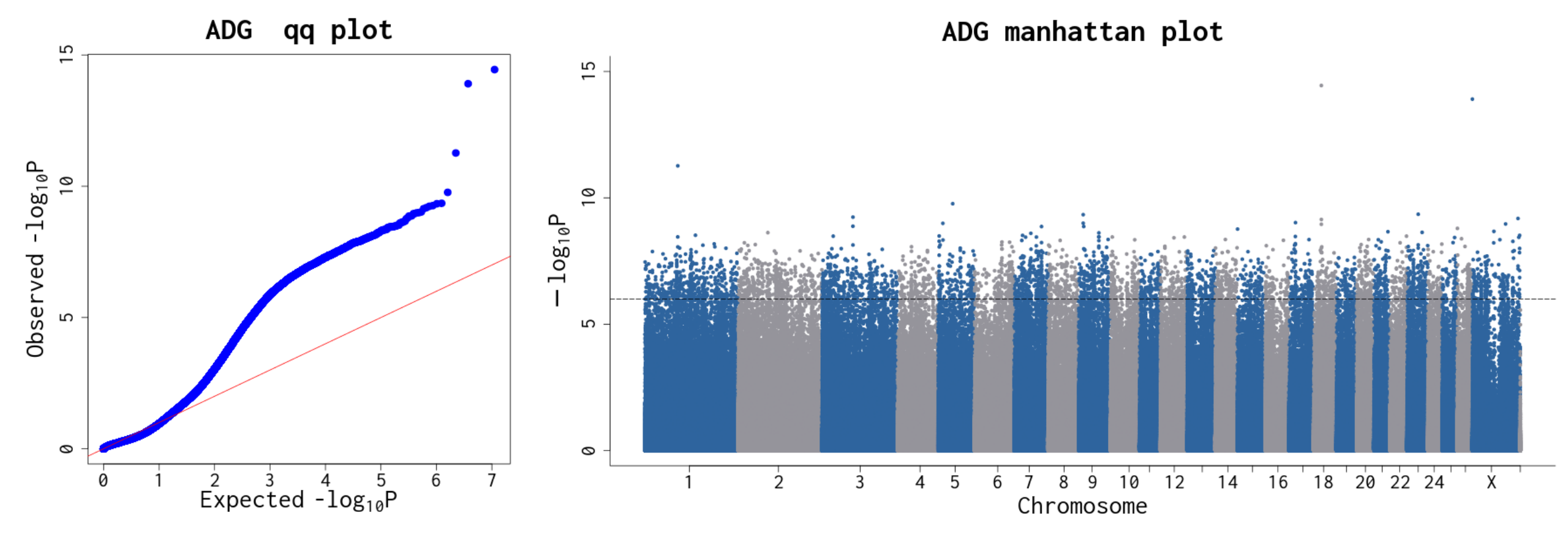

Supplement: Supplementary file 1 [file biology-14-01446-s001.zip › biology-3845291-supplementary/Supplementary Files/Figure S1.png]

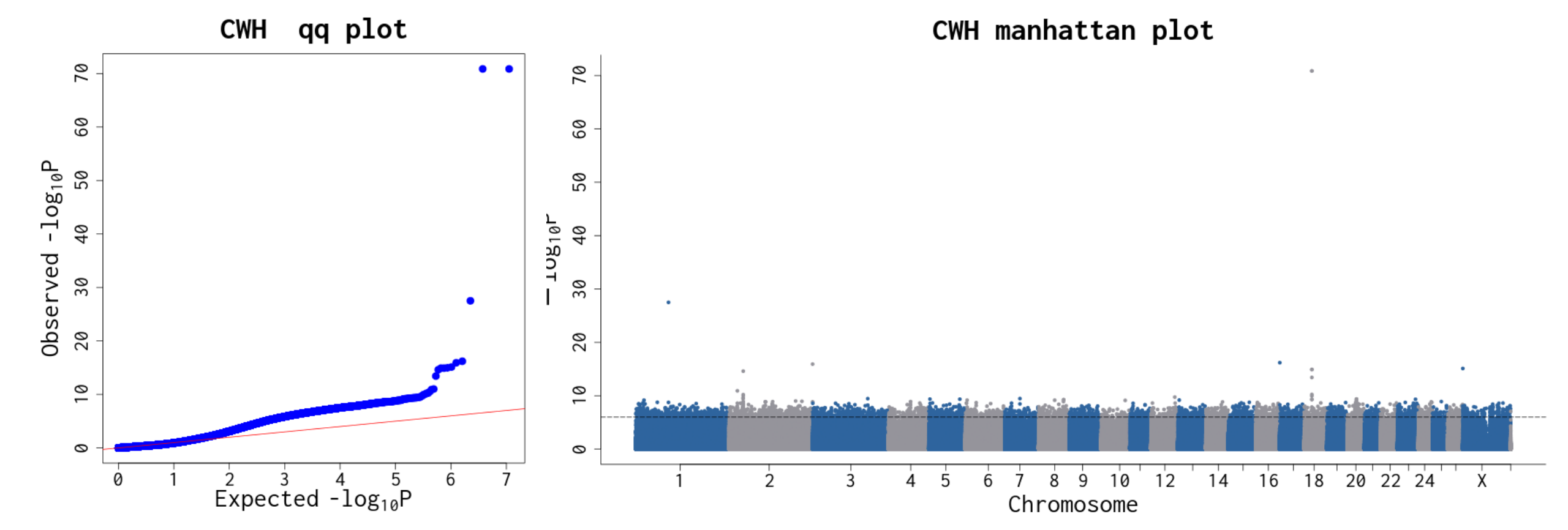

Supplement: Supplementary file 1 [file biology-14-01446-s001.zip › biology-3845291-supplementary/Supplementary Files/Figure S2.png]
